# Mannose Binding Lectin and Susceptibility to Rheumatoid Arthritis in Brazilian Patients and Their Relatives

**DOI:** 10.1371/journal.pone.0095519

**Published:** 2014-04-21

**Authors:** Isabela Goeldner, Thelma L. Skare, Shirley R. Utiyama, Renato M. Nisihara, Hoang van Tong, Iara J. T. Messias-Reason, Thirumalaisamy P. Velavan

**Affiliations:** 1 Department of Medical Pathology, Federal University of Paraná, Curitiba, Brazil; 2 Institute of Tropical Medicine, University of Tübingen, Tübingen, Germany; 3 Rheumatology Unit, Evangelical Hospital, Curitiba, Brazil; 4 Fondation Congolaise pour la Recherche Medicale, Brazzaville , Republic of Congo; University of Leicester, United Kingdom

## Abstract

**Introduction:**

Rheumatoid arthritis (RA) is a commonly occurring systemic inflammatory auto immune disease and is believed to be associated with genetic factors. The innate immune complement protein Mannose binding lectin (MBL) and their *MBL2* genetic variants are associated with different infectious and autoimmune diseases.

**Methods:**

In a Brazilian cohort, we aim to associate the functional role of circulating MBL serum levels and *MBL2* variants in clinically classified patients (n = 196) with rheumatoid arthritis including their relatives (n = 200) and ethnicity matched healthy controls (n = 200). MBL serum levels were measured by ELISA and functional *MBL2* variants were genotyped by direct sequencing.

**Results:**

The exon1+54 MBL2*B variant was significantly associated with an increased risk and the reconstructed haplotype MBL2*LYPB was associated with RA susceptibility. Circulating serum MBL levels were observed significantly lower in RA patients compared to their relatives and controls. No significant contribution of MBL levels were observed with respect to functional class, age at disease onset, disease duration and/or other clinical parameters such as nodules, secondary Sjögren syndrome, anti-CCP and rheumatoid factor. Differential distribution of serum MBL levels with functional *MBL2* variants was observed in respective RA patients and their relatives.

**Conclusions:**

Our results suggest MBL levels as a possible marker for RA susceptibility in a Brazilian population.

## Introduction

Rheumatoid arthritis (RA) is one of the most common inflammatory rheumatic diseases and is closely related to significant increase in healthcare costs and social burden [1]. The early diagnosis and appropriate management of the disease have been established as an effective strategy to minimize the risk of complications and co-morbidities. Nevertheless, tools for RA early diagnosis are still scarce [2]. Genetic factors contribute to at least 60% of the risk of developing this disease [3]–[5] and relatives have an increased risk to develop RA and other autoimmune diseases [6]–[8]. Although extensive research into the pathogenesis of RA, many aspects of the disease are still unclear and the main cause of RA remains unknown.

Complement system is a key component of the innate immunity. The lectin pathway is one of the three pathways of the complement system and can be triggered by pattern-recognition receptors, mainly mannose-biding lectin (MBL), ficolins and collectin 11 [9]. These innate recognitions elements such as MBL and ficolins were associated with susceptibility to various clinical infectious diseases [10]–[14]. These proteins bind to pathogen- or damage-associated molecular patterns and together with MBL-associated serine proteases, activates a cascade of events resulting in membrane attack complex. Although the complement system plays a vital role in pathogen recognition and elimination, there is substantial evidence on its contribution towards immune homeostasis [15]. It recognizes self and nonself antigens and is believed to modulate immunological tolerance towards self antigens avoiding auto immune reactions. Complement system has been related to the development and clinical presentation of many autoimmune diseases [16]–[18].

The etiology of RA is believed to be influenced by genetic and immunological factors. During RA, our own immune system attacks and causes inflammation. The inflammatory process modulates the glycosylation profile of IgG antibodies [19]. Studies have shown that MBL interacts with the IgG G0 glycoform through the exposed GlcNac and thus activating the complement system [20]. Studies have suggested that decreased galactosylation of IgG with high expression MBL2 genotypes are involved in the pathophysiology of RA [21]. Lectin pathway has been involved in the pathology of many rheumatological disorders [22]. In RA, immune complexes are recognized by MBL, leading to complement activation and intense inflammatory response. In this context, higher MBL levels exacerbate complement activation in rheumatic joints, accelerating articular destruction and worsening prognosis [23]. Moreover, high MBL levels have been already related to cardiovascular commitment and premature death in these patients [24]. Interestingly, MBL serum levels were suggested to be elevated in RA patients compared to their first degree relatives, thus revealing their potential significance as a susceptibility marker [25]. On the other hand, low MBL levels have been related to earlier RA development and poor prognosis [26]–[29].

The human MBL encoded on *MBL2* gene is located on chromosome 10. Three single nucleotide polymorphisms (SNPs) in the exon1 of the human *MBL2* gene at codons 52 *(p.Arg52Cys*, MBL2*D), 54 *(p.Dly54Asp*, MBL2*B), and 57 (p.Gly57Glu, MBL2*C), interfere with the formation of higher *MBL* oligomers. These genetic variants modulate the functional activity of the MBL protein and their circulating levels in addition to reduced binding that affects the complement activation [30]–[34]. In addition, two strongly linked SNPs in the proximal promoter (−551 L/H and −221 X/Y), as well as a SNP in the 5′UTR (+4 P/Q); together are linked to three independent non-synonymous SNPs (i.e. MBL2*B, C and *D*) and had been shown to partially account for alterations in complement activation and decreased circulating levels of MBL [35]. In particular, a base substitution at −221 (G to C; promoter allele *X*) is associated with lower MBL serum concentration [36]. Studies have documented that these SNPs contribute to the circulating levels against the seven common secretor haplotypes (namely *HYPA*, *HYPD*, *LXPA*, *LYPA*, *LYPB*, *LYQA* and *LYQC*) [36]. The *HYPA*, *LYQA* and *LYPA* are associated with high expression of MBL protein whereas *LXPA*, *HYPD*, *LYPB* and *LYQC* are associated with low expression of the MBL [37]. The variant alleles have been designated as *O* haplotype, whereas the common *MBL*2 allele is designated as *A* [30]. A total of 24 allelic haplotypes were pre-defined for *MBL2* [38]. Functional MBL deficiency occurs mostly in MBL2*B/B or MBL2*B/C carriers whereas the MBL2*D variant has less influence on MBL structure [32]. The functional *MBL2* alleles and their respective haplotypes have been distributed as different geographical patterns in world populations [39]. Different population specific alleles have shown to contribute to different clinical significance on infectious and auto immune diseases [34]. In the present study, we analyzed the functional role of *MBL2* genetic variants (two strongly linked SNPs in the proximal promoter, one in the 5′UTR and three in exon1) and circulating serum MBL levels and investigate their possible role as a marker for susceptibility and prognosis for RA in a Brazilian cohort.

## Materials and Methods

### Patients

One-hundred and ninety-six (n = 196) adult RA patients were consecutively included from August 2007 till April 2009. All were diagnosed RA according to the American College of Rheumatology (ACR) criteria [40]. Clinical and demographic data were obtained from medical records and interviews using a standard questionnaire (Table 1). Steinbrocker functional classification was applied to determine the extent of physical disability in RA patients. According to this index of disease activity, patients are classified on a four-level scale, ranging from class I (complete functional capacity to carry out all usual duties) to class IV (largely or wholly incapacitated). In the present study, classes III and IV were grouped due to the low number of patients in each representative class. The anti-cyclic citrullinated peptide (anti-CCP) is auto antibody which is used as surrogate markers for diagnosis and prognosis in RA. The Anti-CCP and rheumatoid factor were determined according to standard procedures described elsewhere [6]. Two hundred relatives (n = 200) were also enrolled on this study based on their mutual consent and donated blood samples after their signed consent. Demographic data and articular symptoms suggestive of RA (swollen or tender joints) were investigated using a questionnaire and clinical examination (Table 1). Two hundred healthy matched unrelated individuals (n = 200) from the same geographical area were used as a control group.

**Table 1:** Clinical and demographic features of the RA patients, relatives and healthy controls.

Characteristics	Cases n = 196(%)	Relatives n = 200(%)	Controls n = 200(%)	P value
Age (years)	53 [18–84]	36 [7–91]	46 [24–89]	<0.0001
Gender (Male/Female)	24/132	78/122	40/160	<0.0001
Ethnicity (Afro/Euro/Indian)				NS
*Afro-Brazilian*	113 (72.5)	154 (77.0)	156 (78.0)	
*Euro-Brazilian*	42 (26.9)	46 (23.0)	41 (20.5)	
*Amerindian*	1 (0.6)	0 (0)	3 (1.5)	
Age at disease onset	44 [16–83]	ND	ND	NA
Disease duration	6 [0–60]	ND	ND	NA
Anti-CCP (IU/ml)	110 [6–253]	ND	ND	NA
* Number of positive (≥20)*	152 (77.5)			
* Number of negative (<20)*	48 (24.5)			
Rheumatoid factor (IU/mL)	139 [0–7680]	16 [0–1280]	ND	NA
Functional class (Steinbrock)		ND	ND	NA
* Class I*	90 (45.9)			
* Class II*	79 (40.3)			
* Class III+IV*	27 (13.8)			
Nodules (Yes)^a^	15 (7.6)	ND	ND	NA
Sjögren’s syndrome (Yes)^a^	45 (29.2)	ND	ND	NA
Recurrent infections^b^	25 (33.8)	ND	ND	NA
Articular symptom (swollen or tender joints)	ND	47 (23.5)	ND	NA

Anti-CCP: anti-cyclic citrullinated peptide antibody, (^a^): Total of samples with available data are 154; (^b^): Total samples with available data are 74.

NS: Not significant; NA: not available; ND: not determined. Values expressed in medians and interquartiles range.

Ethnicity was defined according to physical characteristics and informed ethnic background and divided in European; African or Amerindian ancestry. Considering the same ascendency definition, Euro-Brazilians from Southern Brazil have genotype distribution of *MBL2* haplotypes homogeneous with the *MBL2* genotype distribution of most European populations, whereas Afro-Brazilians are similar to eastern Africans [41]. Three ml of venous blood was collected with anticoagulant EDTA. The samples were centrifuged at 800 g for 15 minutes, sera and buffy coat was stored as aliquots at −80°C until used. DNA was extracted from peripheral blood mononuclear cells through DNAzol genomic DNA isolation reagent (Molecular Research Center, Inc., Cincinnati, EUA) according to the manufacturer’s instruction.

### Ethical Statement

Informed written consent was obtained from all study participants or from the parents, whose child was less than 18 years of age. This study was approved by the Ethics Research Committee of the Sociedade Evangélica Beneficiente, Curitiba, Brazil.

### *MBL2* Genotyping

*MBL2* polymorphisms at promoter [−550G/C (H/L), −221G/C (Y/X) and 5′UTR +4C/T (P/Q)] and in exon 1 at codons [52C/T (MBL2*D), 54G/A (MBL2*B) and 57G/A (MBL2*C)] were amplified by PCR and subsequently sequenced utilizing appropriate primers. A 696 bp fragment in the promoter region was amplified using the primer pairs MBL-PromF (5′-GGCCAACGTAGTAAGAAATTTCCAGAGA-3′) and MBL-PromR (5′-GAGGGAGTGATGGAAACAGGGACA-3′). In brief: 2 µl of genomic DNA was amplified in a 20 µl volume of reaction mixture containing 2.5 µl of 10xPCR reaction buffer (20 mM Tris-HCl pH 8.4, 50 mM KCl and 1.5 mM MgCl_2_), 0.2 µl dNTPs 10 mM, 0.5 µl MgCl_2_ 25 mM, 0.25 mM of each primer and 1 U Taq polymerase (QIAGEN). Thermal cycling conditions were 95°C for 4 min; 35 cycles of 95°C for 30 s, 65°C for 30 s, 72°C for 1 min, with a final extension of 72°C for 2 min. Similarly, a 343 bp fragment in the exon 1 was amplified using primer pairs MBL-Ex1F (5′-GTGGCAGCGTCTTACTCAGAAAC-3′) and MBL-Ex1R (5′-TGGGCTGGCAAGACAACTATTAG-3′). Similar cycling conditions as for the promoter fragment amplification were used, except for the annealing temperature which was 61°C for the exon 1 amplification. The amplified PCR fragments were stained with SybrGreen I (Applied Biosystems, Foster City, California, USA) and were visualized on a 1.5% agarose gel. The PCR products were purified by using the illustra GFX PCR and Gel Band Purification kit following manufacturer’s instructions (GE Healthcare, Little Chalfont, UK).

The entire promoter fragment including the 5′ UTR (+4 P/Q) was sequenced with the MBL-PromR and with an internal reverse primer (5′-TCTGCCACCTGAATCCCATCTTTGTATC-3′). A single sequencing reaction was made for the exon 1 fragment with the MBL-Ex1F primer. Purified PCR products were then sequenced with the BigDye Terminator v1.1 Cycle Sequencing Kit (Applied Biosystems, Foster City, CA, USA). Sequencing reactions were analyzed on an automated sequencer (ABI Prism 3130XL Genetic Analyzer, Applied Biosystems). The resulting DNA sequences were aligned using using Codon code Aligner 4.0 software and were reconfirmed visually from their respective electropherograms.

### MBL Levels

MBL serum levels were measured in patients, relatives and healthy control by ELISA as described earlier [42] using monoclonal anti-human MBL antibody HYB131-01 (BioPorto Diagnostics A/S, Copenhagen, Denmark). Individuals with a MBL concentration ≤100 ng/ml were considered MBL low producers or deficient whereas individuals between 100–1000 ng/ml as medium producers and >1000 ng/ml considered as high secretors. The assay cut-off value was 100 ng/ml.

### Statistical Analysis

Direct counting was used to estimate genotype, allele and haplotype frequencies. Tests of independence between RA patients and the comparison group, as well as possible associations between *MBL2* genotypes, alleles or haplotypes and clinical/demographical characteristics were analyzed using logistical regression implicated in Intercooled Stata v 9.2. The odds ratios (OR) and respective *P* values were adjusted for age, gender and ethnicity to exclude the influence of different cofactors to RA susceptibility. The comparison of MBL levels between different groups and the correlations of clinical and demographical characteristics were executed using the SPSS v.19 software by nonparametric Kruskal-Wallis or Mann-Whitney tests and Pearson’s correlation tests, respectively. Deviations from Hardy-Weinberg equilibrium and from the hypothesis of homogeneity between haplotype distributions were tested using the Arlequin software package version 3.1. In all comparisons *P* values less than 0.05 were considered significant.

## Results

### Baseline Characteristics of the Study Cohort

**Figure 1:**
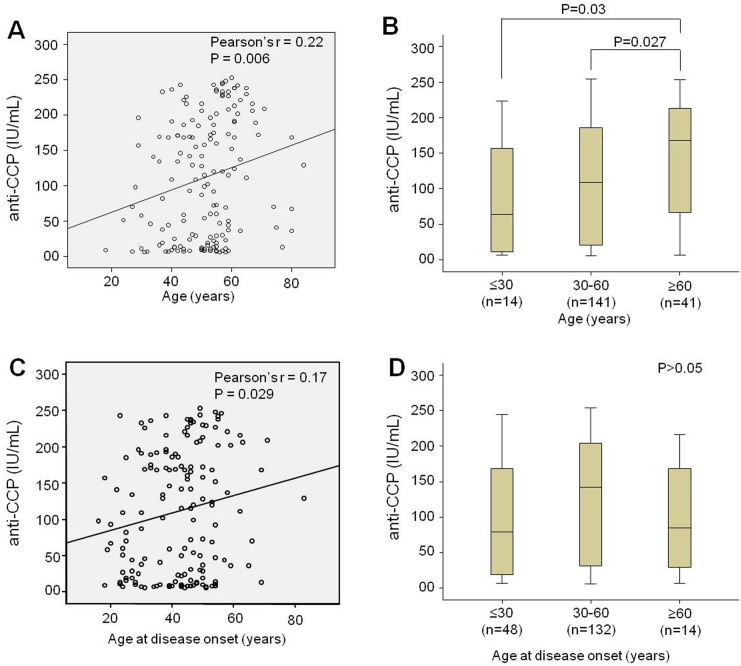
Correlations between anti-CCP and patient’s age and age at disease onset. Anti-CCP correlated with the age of patients (A) and age at disease onset (C). Anti-CCP studied according to patient’s age (B) and age at disease onset (D).

The baseline clinical and demographic characteristics of the RA patients, their relatives and healthy controls are summarized in Table 1. Significant differences were observed in distributions of age, sex and ethnicity in all the three studied groups. The median age at disease onset was 44 (min: 16–max: 83 years) and the median disease duration was 6 years (min: 0–max: 60 years). A significantly weak correlation between anti-CCP levels and the age of patients was observed, of which the elderly patients had higher anti-CCP levels (Pearson’s r = 0.22, P = 0.006). We also observed a significant difference in anti-CCP levels as segregated to different age groups. Additionally, correlation between anti-CCP levels and age at disease onset (Pearson’s r = 0.17, P = 0.024) remained significant. Patients between 30 to 60 years of disease onset had higher anti-CCP levels compared to those with age higher than 60 years (Figure 1). High levels of rheumatoid factor were observed significantly higher in RA patients compared to their relatives. Furthermore, RA patients were also classified based on Steinbrocker, with or without nodules as well as with or without Sjögren’s syndrome (Table 1).

### Functional *MBL2* Variants and RA

The genotype and allele frequencies for the analyzed functional *MBL2* SNPs in patients, relatives and controls were in Hardy-Weinberg equilibrium (P>0.05) except for one variant at codon 54 (rs1800450G/A MBL2*B) in the relatives (P<0.05). The distribution of observed *MBL2* genotype and their respective allele frequencies across different studied variants in different study groups are listed in Table 2. Linkage disequilibrium (LD) pattern of studied *MBL2* variants revealed that variants in the promoter region were in strong LD in RA patients and controls (data not shown).

**Table 2:** Distribution of MBL2 genotypes and alleles in RA patients, relatives and healthy controls.

SNP position	Genotype	Patients	Relatives	Controls	Patients vs. Controls		Relatives vs. Controls		Patients vs. Relatives	
		n = 156(%)	n = 120(%)	n = 200 (%)	OR (95% CI)	*P* value#	OR (95% CI)	*P* value#	OR (95% CI)	*P* value#
**(−221G/C)**										
GG	*YY*	109 (69.9)	91 (75.8)	130 (65)						
GC	*YX*	38 (24.3)	28 (23.3)	58 (29)						
CC	*XX*	9 (8.8)	1 (0.8)	12 (6)						
G	*Y*	256 (82.1)	210 (87.5)	318 (79.5)						
C	*X*	56 (17.9)	30 (12.5)	82 (20.5)		NS	**0.6** **(0.4–0.99)**	**0.047**		NS
Dominant						NS		NS		NS
Recessive						NS		NS		NS
**Exon1 (codon54)**										
GG	*AA*	96 (61.5)	79 (65.8)	148 (74)						
GA	*AB*	55 (35.3)	41 (34.2)	45 (22.5)						
AA	*BB*	5 (3.2)	0	7 (3.5)						
G	*A*	247 (79.2)	199 (82.9)	341 (85.3)						
A	*B*	65 (20.8)	41 (17.1)	59 (14.7)	**1.28** **(1.04–1.6)**	**0.018**		NS		NS
Dominant						NS		NS		NS
Recessive						NS		NS		NS
**Exon1 (codon52+54+57)**										
	*AA*	75 (48.1)	62 (51.7)	119 (59.5)						
	*AO*	73 (46.8)	58 (48.3)	73 (36.5)						
	*OO*	8 (5.1)	0	8 (4)						
	*A*	223 (71.4)	182 (75.8)	311 (77.8)						
	*O*	89 (28.6)	58 (24.2)	89 (22.2)	1.2(1–1.4)	0.058		NS		NS
Dominant					**1.29** **(1.03–1.6)**	**0.026**		NS		NS
Recessive						NS		NS		NS

NS: not significant; NA: not applicable;

# P values were calculated by logistic regression adjusted for age, gender and ethnicity.

Dominant genetic model: Major genotype vs. Heterozygote+Minor genotype; Recessive genetic model: Minor genotype vs. Major+heterozygote genotype.

The minor allele −*221X* in the promoter region was observed significantly higher in the control individuals compared to relatives (OR = 0.6, 95%CI = 0.4–0.99, P = 0.047) whereas the minor allele MBL2*B (codon 54 *rs1800450A*) in exon 1 was observed more frequently in RA patients compared to controls (OR = 1.28, 95%CI = 1.04–1.6, P = 0.018) suggesting an increased susceptibility to RA. In addition, the variant allele *O* of exon 1 was observed to contribute significantly to RA susceptibility in the dominant genetic model (OR = 1.29, 95%CI = 1.03–1.6, P = 0.026) and marginally associated in allelic model (OR = 1.2, 95%CI = 1.0–1.4, P = 0.058). No significant differences were observed for other studied variants in comparisons between the RA patients and controls and/or between relatives and controls. Furthermore, there were no significant differences in genotype and allele frequencies of other studied variants in all comparisons irrespective of patient functional classes (data not shown).

We observed nine secretor haplotypes in our study. The distribution of the reconstructed *MBL2 *secretor haplotypes in the studied cohort is presented in Table 3. The *MBL2* haplotypes were further divided into those associated with high expression (*LYPA*+*LYQA*+*HYPA*) and low expression (*LYQC*+*LXPA+HYPD+LYPB*) of MBL. The *LYPB* haplotype was observed more frequent in RA patients than in controls (OR = 1.28, 95%CI = 1.04–1.6, P = 0.018) suggesting a possible risk factor whereas *LXPA* was observed more frequently in controls compared to relatives (OR = 0.6, 95%CI = 0.4–0.99, P = 0.047). The haplotypes associated with low MBL expression were observed more frequently in RA patients compared to relatives (OR = 1.44, 95%CI = 1.01–2.1, P = 0.038), however no significant distribution was observed after adjusted for age, gender and ethnicity (Table 3).

**Table 3:** Distribution of observed MBL2 haplotype in RA patients, relatives and healthy controls.

*MBL2* Haplotype	Patients	Relatives	Controls	Patients vs. Controls		Relatives vs. Controls		Patients vs. Relatives	
	n = 312(%)	n = 240(%)	n = 400(%)	OR (95% CI)	*P* value#	OR (95% CI)	*P* value#	OR (95% CI)	*P* value#
*HYPA*	80 (25.6)	70 (29.2)	113 (28.3)		NS		NS		NS
*LYPB*	65 (20.8)	41 (17.1)	59 (14.8)	**1.28** **(1.04–1.6)**	**0.018**		NS		NS
*LXPA*	56 (17.9)	30 (12.5)	82 (20.5)		NS	**0.6** **(0.4–0.99)**	**0.047**		NS
*LYQA*	53 (17)	44 (18.3)	79 (19.8)		NS		NS		NS
*LYPA*	26 (8.3)	30 (12.5)	33 (8.3)		NS		NS		NS
*HYPD*	22 (7.1)	15 (6.3)	22 (5.5)		NS		NS		NS
*LYQC*	10 (3.2)	10 (4.2)	9 (2.3)		NS		NS		NS
*LYPD*	0	0	2 (0.5)		NA		NA		NA
*HYPC*	0	0	1 (0.3)		NA		NA		NA
*High expression of MBL*									
*LYPA+LYQA+HYPA*	159 (51)	144 (60)	225 (56.3)		reference		reference		reference
*Low expression of MBL*									
*LYQC+LXPA+HYPD+LYPB*	153 (49)	96 (40)	172 (43)		NS		NS		NS

NS: not significant; NA: not applicable.

# P values were calculated by logistic regression adjusted for age, gender and ethnicity.

### MBL Levels and Rheumatoid Arthritis

**Figure 2:**
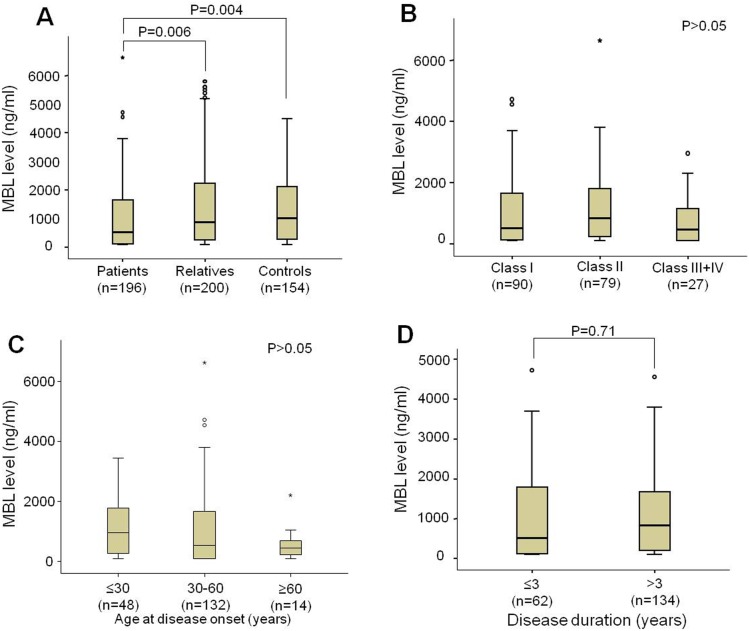
Distribution of MBL levels in studied Brazilian cohort. Distribution of MBL levels in patients, relatives and controls (A), according to different functional classes (B), according to age at disease onset (C) and according to disease duration (D). Open circles indicate possible outliers in each group and the *represents significant distribution amongst the investigated groups.

**Figure 3:**
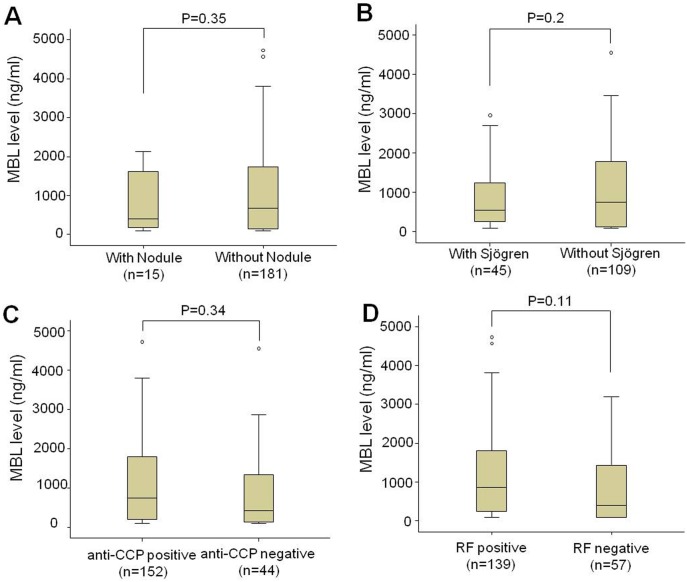
Distribution of MBL levels in RA patients segregated by clinical parameters. Distribution of MBL levels according to presence of nodules (A), Secondary Sjögren’s syndrome (B), positivity for anti-CCP (C) and positivity for Rheumatoid factor (D).

MBL serum levels in RA patients, relatives and controls are presented in Figure 2. RA patients had significantly lower MBL levels compared to relatives and controls (*P* = 0.006 and *P* = 0.004, respectively). No significant differences in distribution were observed between relatives and controls (Figure 2A). MBL levels were higher in the patients with age at disease onset lower than 30 years, and decreased MBL levels in the patients with age at disease onset of more than 30 years (Figure 2C). No significant distributions of MBL levels segregated according to functional classes or disease duration (Figure 2B and 2D) was observed. MBL levels were segregated according to clinical parameters. No significant differences were observed between patients positive and negative for nodules, Sjögren’s syndrome, anti-CCP and rheumaotid factor (Figure 3). Nevertheless, we observed that patients with recurrent infections had lower MBL levels than those without it (*P* = 0.02).

### *MBL2* Variants and MBL Serum Levels in RA Patients

**Figure 4:**
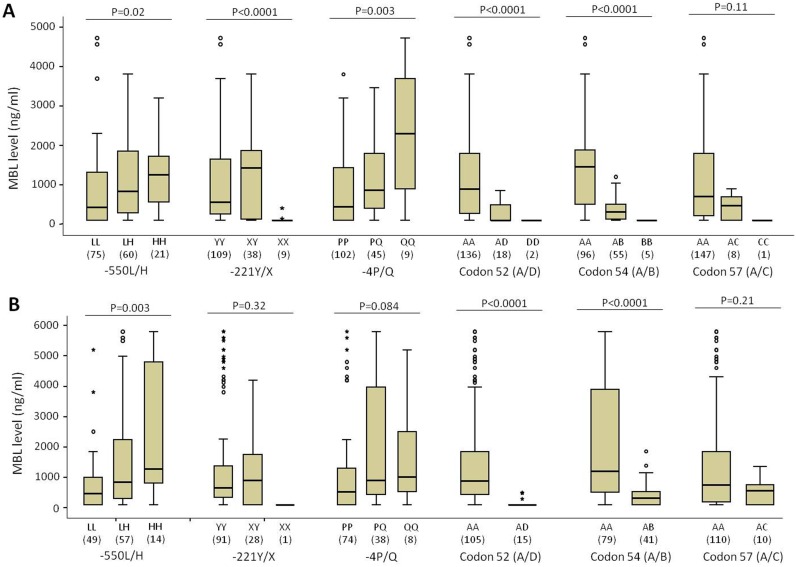
Distribution of MBL levels in different *MBL2* genotypes. Serum MBL levels according to different genotypes of studied *MBL2* variants in RA patients (A) and relatives (B).

The serum MBL levels were observed to segregate according to different *MBL2* genotypes in the investigated cohort. Our results indicate that the presence of minor alleles −*550H* and −*4Q* contributed significantly to higher MBL serum levels whereas minor alleles MBL2*D, MBL2*B and MBL2*C at codons 52, 54 and 57, respectively contributed significantly to lower MBL levels (Figure 4). The homozygous variant genotype −*221XX* presented lower MBL serum levels compared to homozygous wild type genotype −*221YY* and heterozygote genotype −*221XY*. We also observed a similar trend for the effects of *MBL2* variants on MBL levels with relatives (Figure 4).

**Figure 5:**
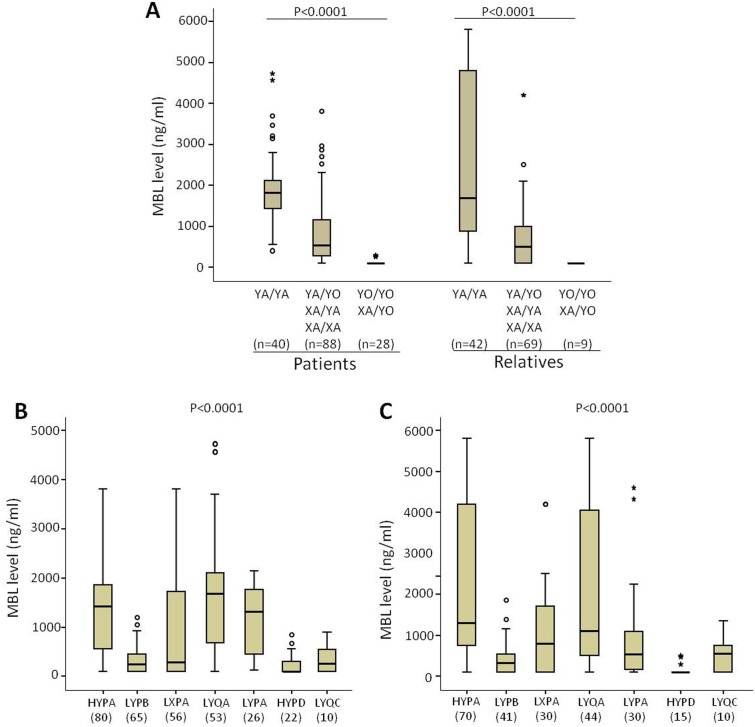
Distribution of MBL levels in different *MBL2* haplotypes and diplotypes. *MBL2* diplotypes were reconstructed from promoter variant −221X/Y and variants in exon1 (Codon 52+54+57, A/O) and divided into high MBL producers (*YA/YA*), intermediate (*YA/YO*, *XA/XA* and *XA/YA*) and low MBL producers (*YO/YO*, *XA/YO*). Serum MBL levels were segregated according to different *MBL2* diplotypes in patients and relatives (A), and different *MBL2 *secretor haplotypes in RA patients (B) and relatives (C).

A base substitution at −221 (G to C; promoter allele *X*) is associated with lower MBL serum concentrations [34]. The *MBL2* diplotypes were reconstructed from promoter variant −221X/Y and variants in exon1 (Codon 52+54+57, A/O) and divided into high (*YA/YA*), intermediate (*YA/YO*, *XA/XA* and *XA/YA*) and low MBL producers (*YO/YO*, *XA/YO*). MBL levels were significantly distributed across high, intermediate and low MBL producers in both RA patients and their relatives (Figure 5A). In addition, the MBL serum levels were significantly distributed across haplotypes in both RA patients and relatives. Patients with *HYPA*, *LYQA* and *LYPA* haplotypes had higher MBL levels compared to other observed haplotypes (Figure 5B and 5C).

## Discussion

Rheumatoid arthritis is an autoimmune disease that involves a complex interplay between environmental and genetic factors. Many immune regulatory factors underlie the initial stage and development of this disease [4], [5], [43]. The activation of innate immune system has been associated with both RA susceptibility and pathophysiology as well as to others autoimmune and infectious diseases [4].

In the present study, we observed that functional *MBL2* variants and MBL levels are significantly associated with RA and equally towards clinical progression in the studied Brazilian cohort. We demonstrated that the allele(s) MBL2*B, the variant allele *O* in the exon 1, the MBL2*LYPB haplotype and lower MBL serum levels as factors for RA development. The association of genetic factors including gene polymorphisms with RA susceptibility has been widely described including *HLA-DRB1* locus, rs2476601 variant in the protein tyrosine phosphatase gene (*PTPN22*), in IL2 receptor genes (rs2104286 in *IL2RA* and rs743777 and *IL2RB*) and in TNF pathway genes such as tumour necrosis factor (*TNF*), alpha-induced protein 2 (*TNFAIP2*) [4], [43]–[46]. Of interest, the innate immune system represented by complement components has been demonstrated to be involved in the development of RA [47]–[49]. Measurements of C5a levels suggested the that complement is activated in rheumatoid joints and it could induce the acute inflammatory process [50]. In RA, the interaction between autoantibodies joint structures, can activate the complement system, triggering inflammatory and adaptive immune response [49]. Mannose-biding lectin (MBL), ficolins and collectin 11 are three major pattern-recognition receptors which initiate the complement system through lectin pathway.

The *MBL2* polymorphisms and MBL serum levels were involved in different infectious diseases such as malaria and schistosomiasis [34], [51], [52] and in autoimmune diseases such as systemic lupus erythematosus and ankylosing spondylitis [20], [53]–[55]. Particularly, the *MBL2* polymorphisms and MBL serum levels have been shown to play an important role in RA susceptibility and its pathophysiology in different populations including Brazil [20], [24], [41], [56]. A previous study, conducted in Brazilian population, showed that genotype *OO* was observed more frequently in the RA patients with rheumatoid nodules [41]. In our study, the alleles MBL2*D, MBL2*B and MBL2*C (allele *O*) that reduces the MBL serum levels was observed to be a risk factor for RA. The secretion profiles of haplotypes observed in our study are in accordance with other published study on RA that has demonstrated the distribution of secretor haplotypes based on MBL levels in a larger Caucasian population of Dutch descent [57]. Recently a meta-analysis investigation has shown that MBL2*B (codon 54) variant is not associated with RA across all published study subjects irrespective of ethnicity, however when stratified by ethnicity in Asian populations, a significant contribution of MBL2*B (codon 54) variant was observed [58].

As described in previously published studies [20], [26], [27], [29], [56], the MBL serum levels were significantly decreased in RA patients compared to controls and in addition no significant difference of MBL serum levels between RA patients and their relatives was observed. A high proportion of RA patients lacked detectable MBL in serum in a longitudinal follow up study and concluded that MBL insufficiency may be a contributing pathogenetic factor in RA [26]. Another chinese study reported that low MBL serum levels predisposes to the development of RA [27]. A yet another study concluded that MBL insufficiency as a significant risk factor for rapid progression of RA [29]. All these studies corroborate our findings that MBL serum levels were significantly decreased in RA patients compared to controls. However a study reported that RA patients had higher MBL levels than their close relatives and controls [25], which was contradictory to the observed findings in this study [25]. High MBL production was associated with an increased overall mortality in RA patients that points to a dual role of this protein in this rheumatic disease [59].

Some studies point to a septic origin of RA and is believed that microbes such as *Porphyromonas gingivalis* are involved in RA pathogenesis that are rich in sugar moieties that are recognized by MBL [60]. We hypothesized, that such pathogens could take advantage of MBL-deficiency and concomitantly induce autoimmune responses against the host. However, this hypothesis must be confirmed by further studies. Considering that RA therapy frequently involves patients immunosuppression, we suggest that MBL levels should be taken to account when choosing the therapeutic strategy.

Although the presence of low producing *MBL2* variants explains the finding of low serum MBL levels, the consumption of this protein during the inflammatory response including in the joints may offer an alternative explanation. As mentioned earlier, complement consumption within the joints has already been described for other complement components, such as C5a [50]. In this situation the reduced MBL levels could also be due to diffusion of this component into the joint cavity favored by increased synovial permeability. Sequestration of complement proteins in synovial cavity has been described to be greater in RA in comparison to other arthropathies [61], [62]. Furthermore, we observed a trend of increased MBL serum levels in the patients with rheumatoid factor and anti-CCP positive in comparison to those with rheumatoid factor and anti-CCP negative. This result suggested that high MBL serum levels regulated by functional *MBL2* variants might possibly affect the pathophysiology of RA. Similar to MBL, ficolins (including ficolin-1, ficolin-2 and ficolin-3 encoded by *FCN1*, *FCN2* and *FCN3,* respectively) are also innate immune recognition proteins that activate complement system [10], [12], [63]. However, only polymorphisms in *FCN1* gene are significantly associated with RA development [64].

Due to the high prevalence of familial RA susceptibility, RA patient’s relatives have also been analyzed. No significant differences in patients and their relatives could be detected in *MBL2* genotype and haplotype frequencies as well as in MBL levels. These results suggest that the *MBL2* functional variants may not be a key genetic factor for RA development, but probably play an inherent cofactor for pathophysiology in RA. Because RA risk is higher in female gender [4] and our study cohort was comprised by diverse ethnicities all the results of the contribution of different cofactors along with *MBL2* polymorphisms to RA susceptibility were adjusted for age, gender and ethnicity. As lower MBL serum levels affects infection’s predisposition [17] this could be an important cofactor for RA susceptibility. More studies in a larger cohort in different world population with more clinical parameters of RA will be essential to validate the role of MBL and other complementary components on the pathophysiology of RA.

In conclusion, our results suggest a significant association of functional *MBL2* polymorphisms and MBL serum levels with RA susceptibility in the Brazilian population. MBL levels may be considered when choosing the therapeutic strategy for RA patients.
